# Does Raising the Arms Modify Head Tremor Severity in Cervical Dystonia?

**DOI:** 10.5334/tohm.623

**Published:** 2021-06-23

**Authors:** Elizabeth Cisneros, Jeanne P. Vu, Ha Yeon Lee, Qiyu Chen, Casey N. Benadof, Zheng Zhang, Emily A. Pettitt, Subhagya K. Joshi, Richard L. Barbano, Joseph Jankovic, Hyder A. Jinnah, Joel S. Perlmutter, Brian D. Berman, Abhimanyu Mahajan, Christopher G. Goetz, Glenn T. Stebbins, Cynthia L. Comella, David A. Peterson

**Affiliations:** 1 Institute for Neural Computation, University of California, San Diego, La Jolla, CA, USA; 2 Department of Neurology, University of Rochester, Rochester, NY, USA; 3 Parkinson’s Disease Center and Movement Disorders Clinic, Department of Neurology, Baylor College of Medicine, Houston, TX, USA; 4 Departments of Neurology and Human Genetics, Emory University, Atlanta, GA, USA; 5 Department of Neurology, Washington University School of Medicine, St. Louis, MO, USA; 6 Departments of Radiology, Neuroscience, Physical Therapy, and Occupational Therapy, Washington University School of Medicine, St. Louis, MO, USA; 7 Department of Neurology, Virginia Commonwealth University, Richmond, VA, USA; 8 Department of Neurological Sciences, Rush University Medical Center, Chicago, IL, USA; 9 Computational Neurobiology Laboratory, Salk Institute for Biological Studies, La Jolla, CA, USA

**Keywords:** head tremor, cervical dystonia, dystonic tremor, posture

## Abstract

**Background:**

A defining characteristic of dystonia is its position-dependence. In cervical dystonia (CD), sensory tricks ameliorate head tremor (HT). But it remains unknown whether raising the arms alone has the same impact.

**Methods:**

We analyzed data collected from patients enrolled by the Dystonia Coalition. For 120 patients with HT, we assessed how raising their arms without touching their head changed their HT severity.

**Results:**

Forty-eight out of 120 patients exhibited changes in HT severity when raising their arms. These patients were more likely to exhibit decreases in HT severity (N = 35) than increases (N = 13, χ^2^ (1, N = 48) = 10.1, p = 0.002). Demographic factors and sensory trick efficacy were not significant predictors of whether HT severity changed when raising their arms.

**Discussion:**

Raising the arms without touching the head is a posture that can reduce HT severity in some CD patients. Our results extend the concept of position-dependent motor symptoms in CD to include the position of the arms.

**Highlights:**

Head tremor (HT) is a prevalent symptom of cervical dystonia (CD) that can often be disabling. This study demonstrates that raising the arms without touching the head is a posture that can reduce HT severity in some CD patients. Our findings also identify a novel form of position-dependence in CD.

## Introduction

Cervical dystonia (CD) is a common form of focal dystonia characterized by muscle spasms that lead to abnormal head posture accompanied by head tremor (HT) in approximately 60% of patients [1,2]. HT is often a disabling aspect of CD that has highly variable responses to conventional treatments [3]. Many patients develop a sensory trick (alleviating maneuver), which is a maneuver, such as touching the cheek, chin, or other area on the face or head, used to normalize abnormal head posture [4]. A sensory trick can also decrease HT severity [5]. Among CD patients with HT who report having a trick, about half exhibit reduced HT severity during execution of the trick while the arms were raised prior to the hand touching the face to execute the trick [5]. This suggests that the widely recognized position-dependent nature of CD motor symptoms [6] includes the position of the arms. In this study, our aim was to determine whether raising the arms alone without touching any part of the head has the potential to decrease HT severity.

## Methods

We analyzed data collected from 206 patients with isolated CD enrolled across 10 sites in the Dystonia Coalition’s previous rating scale validation study (*https://clinicaltri-als.gov/ct2/show/NCT01373424*). All patients provided informed consent prior to their participation in the study. The protocols for original data collection and subsequent analyses were approved by the Human Research Protection Offices at the Washington University School of Medicine (WUSM), Rush University Medical Center (RUMC), and the University of California, San Diego (UCSD; protocol 111255X). All patients were assessed three or more months after their last botulinum neurotoxin (BoNT) injections, and were excluded if they had prior DBS surgery. Patients were on a variety of medications in the following categories: Benzodiazepines, GABAergic drugs (Baclofen, pregabalin), beta adrenergic blockers, and dopamine receptor drugs. We calculated the duration patients had CD by subtracting their reported age of onset from their age at the time the protocol was administered.

Videos of patients were recorded using a standardized examination protocol. The examination included two segments: 1) a baseline segment where patients were instructed to let their head drift to its natural dystonic position, and 2) an “arms up” maneuver segment where patients were instructed to raise their arms into a series of horizontal positions including arms held straight out in pronation, straight out in supination, and in an elbows-bent winged posture. Video segments were annotated using ELAN software version 4.9.4. All video recordings were reviewed by a movement disorders neurologist (CLC) with expertise in dystonia who assessed HT presence and severity during the baseline and arms up segments. During the arms up segment, changes in HT severity were categorized as whether or not they changed by at least an estimated 30% in comparison to the baseline segment, and were scored as decreased, unchanged, or increased.

The examination also included a segment where patients were instructed to demonstrate their most effective sensory trick or, if unaware of a trick, prompted to touch their right cheek, left cheek, and back of their head. The sensory trick was scored using the Toronto Western Spasmodic Torticollis Rating Scale (TWSTRS-2) [7] as complete (0), moderate [1], mild [2], minimal [3], or no improvement of posture [4] by one or more tricks. Because of challenges with multi-rater scoring [2,8], the sensory trick was scored in a separate assessment by the same movement disorders neurologist (CLC), blind to the patients’ original rating.

We tested the null hypothesis that no patients exhibited change in HT with arms up using a binomial test. For those exhibiting change in HT, we determined the likelihood of HT severity increasing or decreasing compared to baseline during the arms up segment using a chi-squared test. For all subsequent analyses, we examined several factors to determine whether or not they were associated with either a change in HT severity and/or whether they were more likely to be associated with an increase or decrease in HT severity. We examined the effect of type of head posture (anterocollis, retrocollis, laterocollis, rotation), the presence of hand tremor, gender, age at onset, and disease duration (and the interaction term between age at onset and disease duration) using a nominal logistic regression. For those patients who reported having a sensory trick, we examined the effect of the trick’s efficacy using a nominal logistic regression. All statistical analysis was performed with SAS’s John’s Macintosh Project (JMP) version Pro 15. We used an alpha level of 0.05 to determine significance.

## Results

Of the 206 patients, 84 were excluded due to the lack of HT, and two were excluded because they were not prompted to perform the arms up segment, leaving 120 patients. Demographics, TWSTRS-2 total motor severity scores, and type of predominant head posture for the patient cohort are provided (***Table 1***).

**Table 1:** Patient Characteristics.


DEMOGRAPHICS	MEAN (SD)

Age at Onset (y)	42.9 (12.3)

Age at Exam (y)	61.1 (9.83)

Disease Duration (y)	18.3 (11.8)

Gender (F/M)	(91/29)

TWSTRS-2 Motor Total	17.4 (5.27)

Range (out of possible 0–48)	3–29

**CERVICAL DYSTONIA POSTURE TYPE**	**COUNT**

Rotation	111

Laterocollis	107

Retrocollis	47

Anterocollis	38


*Note*: SD = standard deviation; TWSTRS-2 = Toronto Western Spasmodic Torticollis Rating Scale; each CD posture type category represents patients that have at least that posture type, however, most patients have a mixture of the four and are counted multiple times.

**Figure 1:**
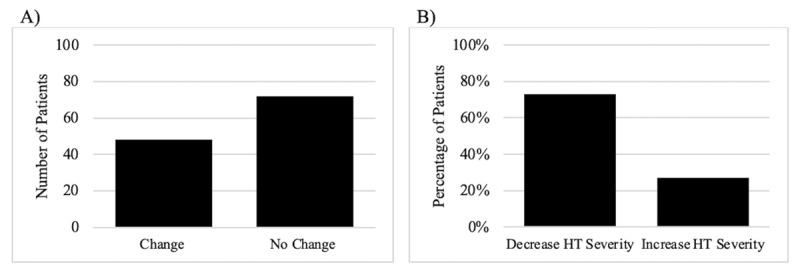
**(A) Frequency of patients that exhibited change or no change in HT severity (B) Percentage of patients that exhibited an increase or decrease in HT severity. (A)** Those with HT are more likely to experience no change in HT severity during the arms up segment. **(B)** Those who exhibited change are more likely to have a decrease in HT severity during the arms up segment.

A proportion of patients significantly greater than zero (48 out of 120) exhibited change in HT severity during the arms up segment (p < 0.0001; ***Figure 1***). Of those that exhibited change in HT severity, it was more likely for them to exhibit a decrease in HT severity (N = 35 patients) than an increase (N = 13, χ^2^ (1, N = 48) = 10.1, p = 0.002; ***Figure 1***).

Type of head posture, the presence of hand tremor, gender, age at onset, disease duration, and the interaction term between age at onset and disease duration were not found to be significant predictors of whether there would be change or no change in HT severity (χ^2^ (9, N = 120) = 4.34, p = 0.887) or an increase or decrease in HT severity (χ^2^ (9, N = 48) = 14.9, p = 0.0953).

Out of 120 patients, 106 reported having a sensory trick. The sensory trick score was not found to be a significant predictor of whether there would be change or no change in HT (χ^2^ (1, N = 106) = 0.08, p = 0.776), or an increase or decrease in HT severity (χ^2^(1, N = 42) = 0.131, p = 0.718).

## Discussion

In this study, we assessed the effect of the arms up postural maneuver on CD HT severity. We found that HT severity changed for 40% of patients with HT. Of those whose HT severity changed with arms up, almost three times as many exhibited decreased HT severity compared to those exhibiting increased HT severity. Our results show that raising the arms alone, without touching the head, is a posture that can reduce HT severity in some patients with CD. Our finding is consistent with the prior observation that about half of CD patients with HT and a sensory trick exhibit reduced HT severity during execution of the sensory trick while the arms were being raised but prior to the hand touching the face [5]. Our findings also extend the widely recognized position-dependent nature of CD motor symptoms [6] to include the position of the arms. We also found that sensory trick efficacy was not a significant predictor of the effect of arms up on HT severity. Although tricks and raising the arms share elements of proprioceptive dynamics, our results suggest that they have at least partly independent influences on CD motor symptoms.

It remains unclear *how* the arms up maneuver reduces HT severity. It could be due to the role of proprioception, motor programming, biomechanical interactions, or some interaction thereof. Proprioceptive sensory input changes when raising the arms. Patients with CD have abnormal head proprioception evidenced by abnormal response to neck muscle vibration [9]. In addition, while performing out-of-sight upper limb reaching movement towards a target, CD patients showed significant trajectory abnormalities in comparison to normal controls [10]. One study showed that although tactile abnormalities were present in CD patients with or without tremor, proprioceptive dysfunction was observed in only those with tremor [11]. Motor programming has been suggested as a potential mechanism for the sensory trick because an effective sensory trick relates to not only increased parietal and bilateral visual cortex activation but also decreased activation in contralateral supplementary motor area and primary sensorimotor cortices [12]. Finally, biomechanical interactions between muscles involved in raising the arms and those producing head tremor may also play a role in how raising the arms decreases HT severity. Raising the arms involves levator and trapezius muscles, which are also implicated in head positioning. Although the arms up maneuver and sensory tricks share elements of upper limb proprioception, motor programming, and biomechanics, the arms up maneuver in our study has not been documented as a self-initiated maneuver that patients use and is therefore not considered a conventional sensory trick.

This study has a few limitations. First, we measured changes in HT severity categorically instead of numerically. More precise measures of HT severity may be more sensitive for detecting these changes. Second, we do not have data on how the sensory trick affected the severity of HT and head posture separately or how the arms up maneuver affected head posture. This information could be used to determine whether the arms up maneuver and sensory tricks have differential effects on HT even for individual patients. Third, although in our cross-sectional design we found that the likelihood that raising arms decreases HT severity is independent of demographic variables, HT likelihood in CD is higher for older age at onset [13,14], longer disease duration [14,15], and female gender [1,14]. Longitudinal studies could determine whether individual patients exhibit changes over time in the relationship of HT to the arms up maneuver.

Future studies on the role of proprioception and motor programming in CD could benefit from experimental designs and analyses that go beyond this study. In terms of experimental design, one could passively move patients arms up or have them imagine moving their arms up. This would help determine if, like the sensory trick, imagined motor programming can modulate HT severity. In terms of new analyses, more fine-grained methods to capture change in HT – such as with computer vision-based software that can quantify HT severity from videos – will also help us determine how proprioception and motor programming influence their CD symptoms.

## Data accessibility statement

Original patient data available from the Dystonia Coalition upon reasonable request.
